# Effects of Dietary Andrographolide Levels on Growth Performance, Antioxidant Capacity, Intestinal Immune Function and Microbioma of Rice Field Eel (*Monopterus Albus*)

**DOI:** 10.3390/ani10101744

**Published:** 2020-09-25

**Authors:** Yong Shi, Lei Zhong, Yanli Liu, Junzhi Zhang, Zhao Lv, Yao Li, Yi Hu

**Affiliations:** 1 Hunan Engineering Research Center for Utilization of Characteristics of Aquatic Resources, Hunan Agricultural University, Changsha 410128, China; 15570877892@163.com (Y.S.); zhonglei-5@163.com (L.Z.); 18390940935@163.com (Y.L.); junzhizh050816@163.com (J.Z.); 18012481396@163.com (Z.L.); Yao.li11@163.com (Y.L.); 2 Hunan Key Laboratory of Traditional Chinese Veterinary Medicine, Hunan Agricultural University, Changsha 410128, China

**Keywords:** andrographolide, *Monopterus albus*, growth, antioxidant capacity, intestinal immune, intestinal microbioma

## Abstract

**Simple Summary:**

This study investigated the effects of dietary andrographolide on the growth performance, antioxidant capacity, intestinal immune function and microbioma of rice field eel. This study indicated that the diets supplemented with low-dose andrographolide (75 and 150 mg/kg) significantly improved growth performance, enhanced antioxidant capacity and regulated the intestinal physical barrier and microbiota of *M. albus*. In addition, dietary supplementation of andrographolide upregulated of anti-inflammatory cytokines and downregulated of proinflammatory cytokines. The anti-inflammatory function of andrographolide may be related to the suppression of the toll-like receptor signaling pathway. These results can provide the valuable data for future rice field eel feeds.

**Abstract:**

An eight-week feeding trial was conducted to investigate the effects of dietary andrographolide on the growth performance, antioxidant capacity in the liver, intestinal inflammatory response and microbiota of *Monopterus albus*. A total of 900 health fish (25.00 ± 0.15 g) were randomly divided into five groups: AD1 (the basal diet) as the control, and AD2, AD3, AD4 and AD5 groups, which were fed the basal diet supplemented with 75, 150, 225 and 300 mg/kg andrographolide, respectively. The results showed that compared with the control group, dietary andrographolide supplementation (1) significantly increased trypsin and lipase activities in the intestine, and increased the weight gain rate but not significantly; (2) significantly increased the levels of glutathione reductase (GR), glutathione (GSH) and glutathione peroxidase (GPx) and the content of in the liver; significantly decreased the contents of reactive oxygen species (ROS) and malondialdehyde (MDA); remarkably upregulated the Nrf2, SOD1, GSTK and GSTO mRNA levels in the liver; downregulated the Keap1 mRNA level; (3) significantly increased the villi length and goblet cell numbers in the intestine, remarkably upregulated the Occludin mRNA level in the intestine, downregulated the Claudin-15 mRNA level; (4) remarkably upregulated the IL-10, TGF-β1 and TGF-β3 mRNA levels in the intestine; downregulated the IL-12β and TLR-3 mRNA levels; (5) significantly decreased the richness and diversity of the intestinal microbioma, increased the percentages of Fusobacteria and Firmicutes and significantly decreased the percentages of Cyanobacteria and Proteobacteria. In conclusion, these results showed that dietary low-dose andrographolide (75 and 150 mg/kg) promoted growth and antioxidant capacity, regulated the intestinal microbioma, enhanced intestinal physical and immune barrier function in rice field eel.

## 1. Introduction

With the rapid expansion of the scale of aquaculture and the increasing degree of intensification as well as the inappropriate use of feed ingredients, the immunity and intestinal health of fish are being adversely affected [1]. In the past, to prevent and control the aquatic animal diseases, antibiotics were commonly used in aquatic feed. However, due to drug resistance, drug residues and water pollution, their applications have been restricted [2]. In addition, The European Union began to restrict the use of feedstock antibiotics in 2006, and China has strengthened the management and application of antibiotics in recent years. Therefore, there is an urgent need to search for alternative strategies to increase disease resistance for the development of antibiotic-free, sustainable aquaculture [3,4].

In recent years, effective components derived from plants and herbs have often been considered as an alternative eco-friendly feed additive strategy in aquaculture [5]. Many studies have revealed their beneficial effects in terms of promoting the growth, immunity and disease resistance of economic fish, such as *Mucuna pruriens* and *Withania somnifera* in rohu (*Labeo rohita*) [6,7], sanguinarine (*Macleaya cordata*) in koi carp (*Cryprinus carpiod*) [8], astragalus polysaccharide (*Astragalus*) in pacific white shrimp (*Litopenaeus vannamei*) [9], aloe vera in pacu (*Piaractus mesopotamicus*) [10] and katuk (*Sauropus androgynus L. Merr.*) in namilton (*Epinephelus coioides*) [11]. Andrographolide, a diterpenoid, is the main active ingredient of *Andrographis paniculata*, and its structural formula is C_20_H_30_O_5_ [12,13]. It is mainly concentrated in the leaves and can be easily separated from crude plant extracts [14]. A large amount of evidence has shown that andrographolide exhibits a wide range of biological activities such as anti-inflammatory [15,16], antibacterial [17], anticancer [18], antioxidant [15], antipathogenic microorganism [19], anticardiovascular disease [20] and liver- and gallbladder-protective properties [21]. In aquatic animals, the beneficial effects of dietary andrographolide have only been verified in *Labeo rohita* fingerlings [22].

Rice field eel (*Monopterus albus*) is an important freshwater breeding species in China, and its production is currently estimated to be close to 320,000 tons, with a value of 20 billion yuan in 2019 [23]. Due to the deterioration of the breeding environment and the deterioration of feed quality, the immunity of rice field eel is reduced [24,25]. Toll-like Receptors (TLRs) are a kind of important receptor of pathogen recognition molecules, and are bridges of innate and acquired immunity of the body [26]. Studies have shown that the TLR signaling pathway is involved in intestinal inflammation [27]. In addition, the intestinal microbioma has made significant contributions to the health of their hosts. Studies have shown that the intestinal microbioma has important relationships with metabolic activities, feed conversion, immunity and disease resistance [28]. However, the effect of dietary andrographolide on rice field eel has not been reported. Therefore, the overall objective of this study was to evaluate the effects of dietary andrographolide on growth performance, antioxidant response in the liver, intestinal microbioma and intestinal immune function in rice field eel. The findings of this study will provide a clue for the application of andrographolide in aquatic animals feed.

## 2. Materials and Methods

### 2.1. Preparation of Experimental Diets and Experimental Design

In this experiment, the basal diet was formulated to contain 43.16% crude protein and 5.16% crude lipid. The protein and lipid sources were the same as our previous study [29]. Five experimental diets were supplemented with andrographolide (Hunan Jiaruisi Biological Technology Co., Ltd. Changsha, China) at the graded levels of 0 (control, AD1), 75 (AD2), 150 (AD3), 225 (AD4) and 300 mg/kg (AD5) (Table 1). The purity of andrographolide was greater than 98% according to area normalization of high-performance liquid chromatography. The main chromatographic conditions included: Column: shim-pack CLC-ODS (150 mm × 6 mm, 5 μm); mobile phase: methanol-water (60:40); flow rate: 1.0 mL/min; column temperature: 40 °C; detection wave length: 254 nm. Andrographolide was added to the feed in the form of powder. The processing, storage and feeding of the feed stuffs were the same as our previous study [29]. *M. albus* were reared in floating net cages (1.5 × 2 × 1.5 m). The cages were made up of polyamides with a mesh size of 0.25 mm. The water exchange rate of pond is about 15–20 m^3^/h.

Source and acclimation of *M. albus* fingerlings were referred to in our previous reports [29,30]. After the acclimation period, health fingerlings (25.00 ± 0.15 g) were randomly distributed into 15 floating cages, with each cage containing 60 fish per replicate. Each group (in triplicate) was fed one of the diets with different levels of andrographolide, as follows: control, AD1 (without andrographolide); AD2 (75 mg/kg); AD3 (150 mg/kg); AD4 (225 mg/kg); and AD5 (300 mg/kg), for a period of 56 days. The fish were fed at a rate of 3–5% of body weight once a day at 17:00–18:00. We usually adjusted the feed amount every five days according to the fish growth. We generally adjusted the feed amount every five days, and estimate the weight gain every five days according to the feed conversion rate of 1.5. The water temperature (28.3 ± 2.6 °C), pH (7.2 ± 0.5), dissolved oxygen (6.5 ± 0.3 mg/L), ammonia nitrogen (0.46 ± 0.03 mg/L) and natural light were kept stable during the experimental period.

### 2.2. Sample Collection

All experiments were performed in accordance with the European Union regulations concerning the protection of experimental animals. Pretreatment, anaesthesia and anatomy of the experimental *M. albus* before sampling and the methods of sample collection were referred to in our previous report [29]. The middle intestines from three *M. albus* were removed and immersed in 4% paraformaldehyde to make intestinal slices. At the same time, the posterior intestine and liver from three *M. albus* were pooled into 1.5 mL tubes and then stored at −20 °C for further analysis. The intestinal contents of the posterior intestines of six fish from each cage were collected, and the samples from three fish were mixed together and stored at −80 °C until further analysis. The posterior intestine and liver of three fish from each cage were collected and mixed together in 1.5 mL RNase-free tubes, respectively, immediately stored in liquid nitrogen and then transferred to −80 °C refrigerator for about a week for sample homogenization to isolate RNA.

### 2.3. Determination of Growth Parameters

The survival rate (SR), weight gain rate (WGR), feed conversion ratio (FCR), condition factor (CF), hepatosomatic index (HSI) and viserosomatic index (VSI) were calculated according to our previous report [24].

Survival rate (SR, %) = N_f_/N_i_ × 100 (1)

Weight gain rate (WGR, %) = (W_t_ − W_o_)/W_o_ × 100 (2)

Feed conversion rate (FCR) = total amount of the feed consumed (g)/(W_t_ − W_o_) (3)

Condition factor (CF, g/cm^3^) = W_t_ × 100/(body length)^3^ (4)

Hepatosomatic index (HSI, %) = liver weight (g)/W_t_ × 100 (5)

Viserosomatic index (VSI, %) = visceral weight (g)/W_t_ × 100 (6)

where N_f_ and N_i_ are the final and initial numbers of fish, respectively; and W_t_ (g) and W_o_ (g) are the final and initial fish weights, respectively.

### 2.4. Measurement of Digestive Enzyme Activities and Liver Antioxidant Parameters

Posterior intestine and liver samples were rinsed with 0.70% physiological saline and then homogenized on ice with 9 volumes (v/w) of cold physiological saline. Then centrifuge at 3500×g for 10 min at 4 °C to collect the supernatant and analyze the following parameters. The total protein content was determined by bisindoleacetic acid (BCA) method. The activities of trypsin, lipase and amylase in the posterior intestine were determined by a commercial kit (Nanjing Jiancheng Bioengineering Institute, Nanjing, China) [29]. The trypsin activity was spectrophotometrically determined with arginine ethyl ester as a substrate determined at 253 nm. The lipase activity was enzyme colorimetry measured at 580 nm. The amylase activity was Iodine-starch colorimetry measured at 660 nm.

Reactive oxygen species (ROS) levels were measured by using the enzyme-linked immunosorbent assay (ELISA) kit. An ROS ELISA kit was purchased from ZciBio Technology Co., Ltd., Shanghai, China. Determination principle: The purified ROS capture antibody was coated with a microporous plate to make a solid phase antibody. Samples were successively added to the coated micropores, and then combined with HRP-labeled detection antibodies to form an antibody–antigen–enzyme-labeled antibody complex. After thorough washing, substrate 3,3,5,5-TetraMethyl benzidine (TMB) was added for color development. TMB was converted to blue under the catalysis of HRP enzyme, and finally to yellow under the action of acid. The absorbance was measured with an enzyme marker at the wavelength of 450 nm [31]. The ROS level was calculated according to the measured standard curve. Glutathione (GSH) and malondialdehyde (MDA) contents and activities of glutathione S-transferase (GST), glutathione reductase (GR), superoxide dismutase (SOD), catalase (CAT) and glutathione peroxidase (GPx) in liver were assayed by using a commercial kit (Nanjing Jiancheng Bioengineering Institute, Nanjing, China). The GSH was reacted with dithionitrobenzoic acid, and the GSH content was quantitatively determined by a colorimetric method at 405 nm. The SOD activity was water soluble tetrazolium (WST-1) measured at 450 nm. The GST and GPx activities were spectrophotometrically measured at 412 nm. Additionally, the GR, CAT activities and MDA content were spectrophotometrically measured at 340, 405 and 532 nm, respectively.

### 2.5. Intestinal Histological Structure

The samples were immediately fixed in paraformaldehyde solution, and slides were prepared by subjecting the samples to washing, dehydration using different grades of alcohol, clearing with xylene and embedding in paraffin wax. The wax blocks were sectioned to a thickness of five microns and stained with haematoxylin and eosin (H&E). Villus length, muscular thickness and goblet cell numbers were measured according to Ramos et al. [32].

### 2.6. Reverse Transcription Real-Time Fluorescent Quantitative Polymerase Chain Reaction (RT-qPCR) Analysis

Total RNA from the posterior intestine and liver was extracted using TRIzol reagent (Invitrogen, Carlsbad, CA, USA) following the manufacturer’s protocol. A 30–50 mg sample was added to 1 mL TRIzol for homogenization (6000 rpm, 30 s), followed by 0.2 mL chloroform, which was violently oscillated for 15 s, and centrifuged at 12,000 rpm at 4 °C for 15 min. The upper layer of 500 μL colorless aqueous phase was absorbed, isopropyl alcohol was added in the same volume, and centrifuged at 12,000 rpm at 4 °C for 10 min. Wash with 75% ethanol and centrifuge at 12,000 rpm at 4 °C for 3 min. The supernatant was then removed and dried. RNA was dissolved in 50 μL enzyme-free water. The RNA samples were analyzed by 1.5% agarose electrophoresis and quantitated at 260 nm with a NanoDrop ND-2000 UV-Visible Spectrophotometer. All OD260/OD280 values were between 1.8 and 2.0 [33]. First strand cDNA was synthesized from 1 µg total RNA using Reverse Transcriptase MMLV Kit (Takara, Dalian, China) following the manufacturer’s protocol. The first-strand cDNA was stored at −80 °C. The RT-qPCR was carried out with a Bio-Rad CFX96 system (USA) with SYBR Premix Ex TaqⅡ (TaKaRa, Dalian, China). The total 25 μL volume of the PCR reaction was composed of 12.5 μL SYBR Premix Ex TaqⅡ (2×), 1 μL forward primer, 1 μL reverse primer, 2 μL cDNA and 8.5 μL sterile double-distilled water [34]. The program was 95 °C for 30 s followed by 35 cycles of 95 °C for 5 s, 58 °C for 15 s and 72 °C for 20 s. Melting curve analysis of PCR products was performed at the end of each PCR reaction to confirm the specificity. The mRNA primer sequences are listed in Table 2, and RPL-17 was selected as a reference gene. Expression was calculated using the Ct (2 ^−ΔΔCt^) method [35,36,37].

### 2.7. Intestinal Microbiology

According to the growth performance, the AD1, AD2, AD3 and AD5 groups were selected for intestinal microbioma analysis. High-throughput sequencing was performed using the Illumina MiSeq platform, with the amplification of the 16S rRNA V3-V4 region. All sequences were classified into operational taxonomic units (OTUs), selected at a 97% similarity level using QIIME (version 2.0, http://qiime.org/index.html) after the removal of low-quality scores (Q score, 20) with a FASTX-Toolkit (Hannon Lab, NY, USA) [38]. Six indices (the observed species, Chao1, Shannon, PD whole tree, ACE and Good‘s coverage indices) that were used to analyze the complexity of species diversity in a sample were calculated using QIIME (Version 2.0, http://qiime.org/index.html) and displayed with R software (Version 2.15.3) [39]. Beta diversity analysis was performed to investigate the structural variation of the microbial communities across samples using UniFrac distance metrics [40], and the principal coordinates analysis (PCoA) was also performed using QIIME (version 2.0, http://qiime.org/index.html). Differences in the UniFrac distances for pairwise comparisons between groups were determined using a Student’s t-test and the Monte Carlo permutation test with 1000 permutations [29]. The results were visualized in box-and-whisker plots. Taxon abundance at the phylum and genus levels were statistically compared between groups using the Metastats program.

### 2.8. Statistical Analysis

All statistical analyses were performed using SPSS 24.0 (SPSS Inc., Michigan Avenue, Chicago, IL, USA). Growth performance, enzyme activities, intestinal morphology and mRNA levels were carried out using nonparametric tests. The nonparametric Kruskal–Wallis rank sum test and posthoc pairwise comparisons using the Mann–Whitney U test (*p*-values were adjusted using the Benjamini–Hochberg correction) were performed. A significance level of *p* < 0.05 was chosen.

Alpha-diversity indices (observed species, Chao1, Shannon, PD whole tree, ACE and Good‘s coverage) were checked for normality and homogeneity of variance by Shapiro–Wilk and Levene tests, respectively. These data were compared by one-way analysis of variance (ANOVA), and differences between the means were tested by Duncan’s multiple-range test. All results are reported as the “mean ± S.E.”, and differences were considered significant at *p* < 0.05 [41]. In addition, the overall difference in the bacterial community was evaluated by analysis of similarity (ANOSIM) [42].

## 3. Results

### 3.1. Growth Performance

**Figure 1:**
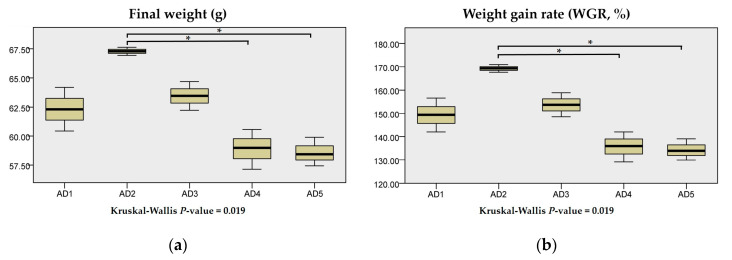

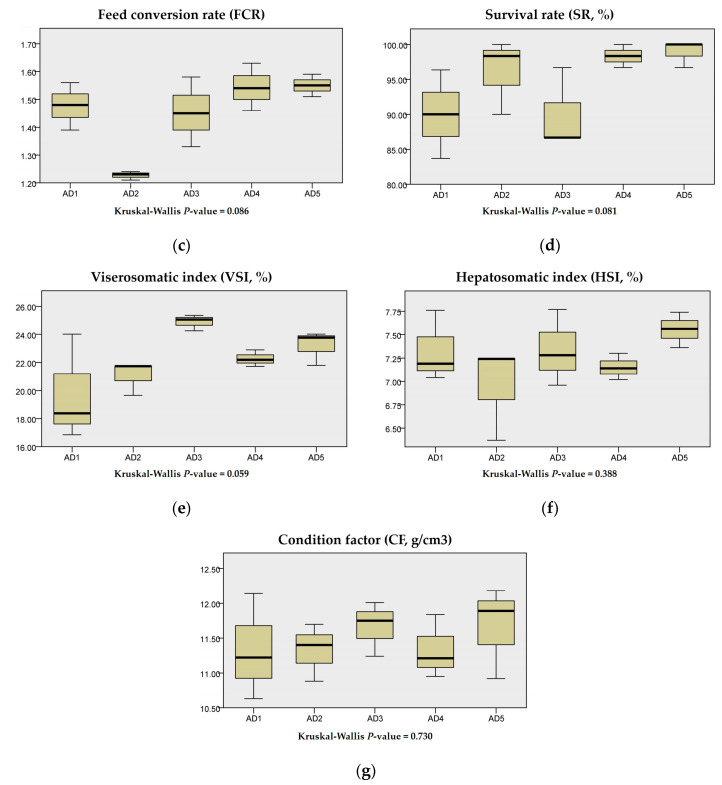
Effects of dietary andrographolide on growth performance of rice field eel, *Monopterus albus*. (**a**), (**b**), (**c**), (**d**), (**e**), (**f**) and (**g**) are final weight, weight gain rate (WGR), feed conversion rate (FCR), survival rate (SR), viserosomatic index (VSI), hepatosomatic index (HSI) and condition factor (CF), respectively. Note: * *p* < 0.05.

FCR, SR, HIS, VSI and CF showed no significant differences between the treatments (*p* > 0.05) (Figure 1). The final weight and WGR were higher in the AD2 group than those in the AD1 group but not significant (*p* > 0.05). The final weight and WGR in the AD2 group were significantly higher than those in the AD4 and AD5 groups (*p* < 0.05). FCR in the AD2 group was decreased compared to those of other groups (AD1, AD3, AD4 and AD5) but not significant (*p* > 0.05).

### 3.2. Digestive Enzyme Activities in Intestine

**Figure 2:**
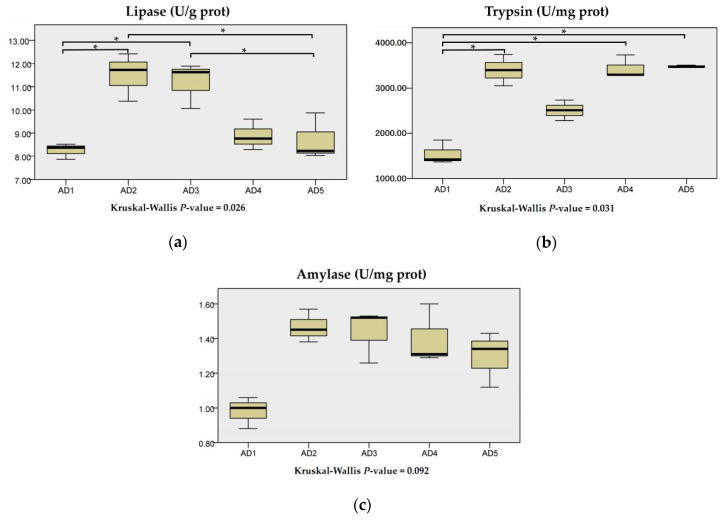
Effects of dietary andrographolide on intestinal digestive enzymes of rice field eel, *Monopterus albus*. (**a**), (**b**) and (**c**) are lipase, trylase and amylase, respectively. Note: * *p* < 0.05.

As shown in Figure 2, amylase activity in the intestine showed no significant differences between the treatments (*p* > 0.05). Trypsin activity in the intestine was significantly increased when the eels were fed a diet supplemented with 75, 225 and 300 mg/kg andrographolide compared to the AD1 group (*p* < 0.05). Lipase activity in the AD2 and AD3 groups was significantly higher than that in the AD1 and AD5 groups (*p* < 0.05).

### 3.3. Antioxidant Enzyme Activities in the Liver

**Figure 3:**
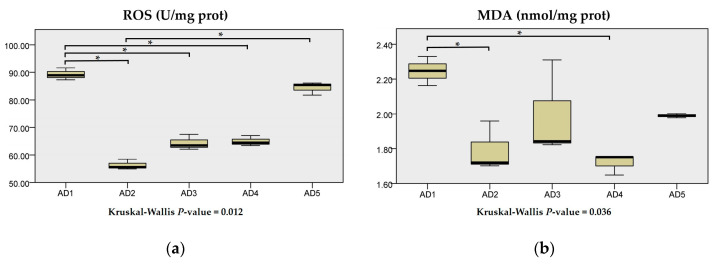
Effects of dietary andrographolide on reactive oxygen species (ROS) (**a**) and malondialdehyde (MDA) (**b**) in the liver of rice field eel, *Monopterus albus*. Note: * *p* < 0.05.

The content of MDA in the AD2 and AD4 groups, and the content of ROS in the AD2, AD3 and AD4 groups were significantly decreased compared to the AD1 group (*p* < 0.05; Figure 3).

**Figure 4:**
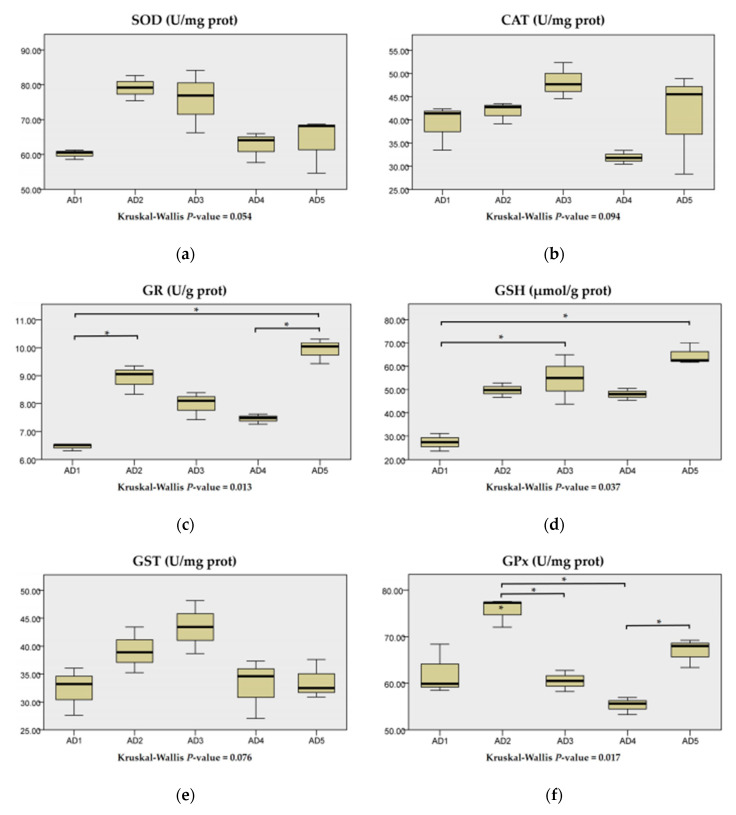
Effects of dietary andrographolide on antioxidant indexes in the liver of rice field eel, *Monopterus albus*. (**a**), (**b**), (**c**), (**d**), (**e**) and (**f**) are superoxide dismutase (SOD), catalase (CAT), glutathione reductase (GR), glutathione (GSH), glutathione S-transferase (GST) and glutathione peroxidase (GPx), respectively. Note: * *p* < 0.05.

As shown in Figure 4, the activities of SOD, CAT and GST in the liver were higher in the AD2 and AD3 groups than those in the AD1 group but not significant (*p* > 0.05). Compared to the AD1 group, the activities of GR in the AD2 and AD5 groups, and the contents of GSH in the AD3 and AD5 groups, were significantly increased (*p* < 0.05). The GPx activity in the AD2 group was significantly higher than that in the AD3 and AD4 groups (*p* < 0.05).

### 3.4. Antioxidant Related Gene Expression in the Liver

**Figure 5:**
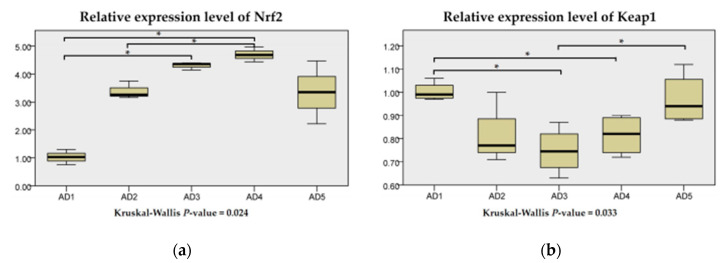
Relative expression levels of Nrf2 (**a**) and Keap1 (**b**) in the liver of rice field eel (*Monopterus albus*) fed diets containing graded levels of andrographolide. Note: * *p* < 0.05.

Compared to the AD1 group, the expression level of Nrf2 was significantly upregulated (*p* < 0.05) in eels fed a diet supplemented with 150 and 225 mg/kg andrographolide groups. The expression level of Keap1 in the AD3 and AD4 groups was significantly lower than that in the AD1 group (*p* < 0.05; Figure 5).

**Figure 6:**
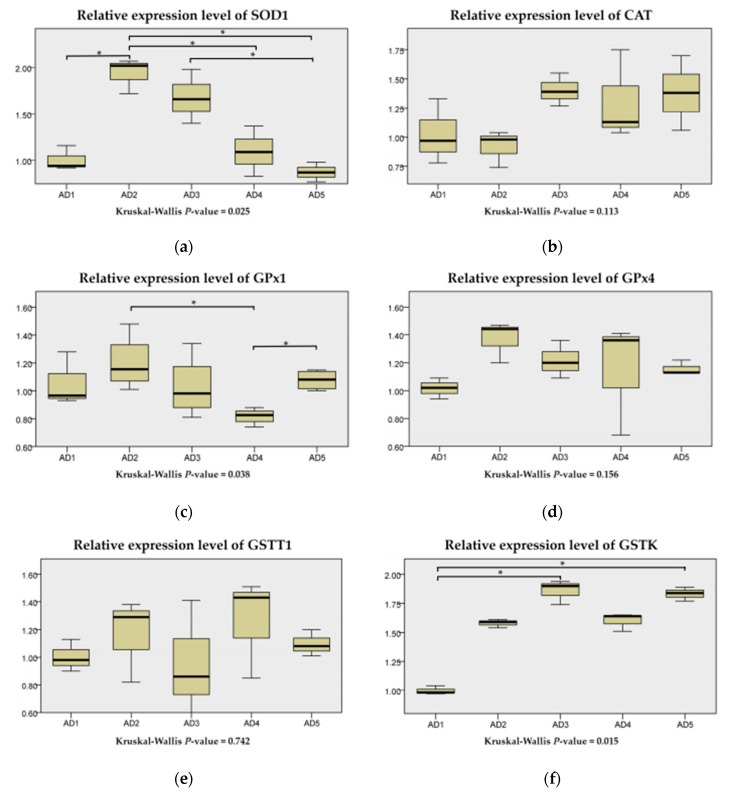

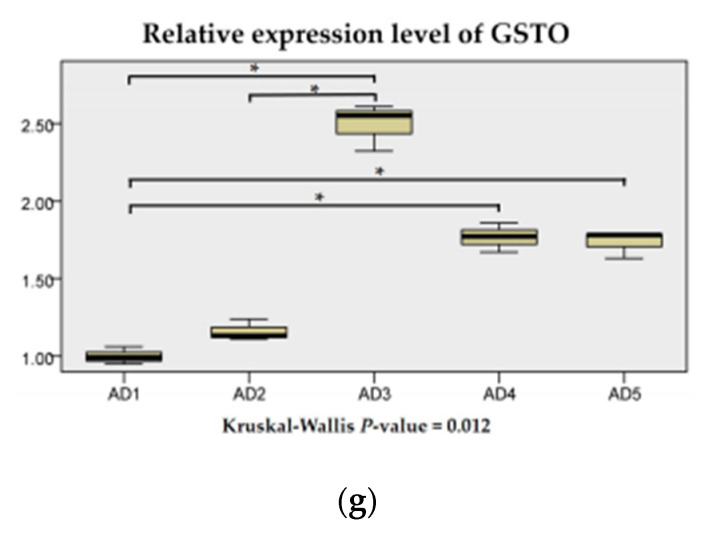
Relative expression levels of SOD1 (**a**), CAT (**b**), GPx1 (**c**), GPx4 (**d**), GSTT1 (**e**), GSTK (**f**) and GSTO (**g**) in the liver of rice field eel (*Monopterus albus*) fed diets containing graded levels of andrographolide. Note: * *p* < 0.05.

As shown in Figure 6, compared to the AD1 group, the mRNA level of SOD1 in the AD2 group; the mRNA levels of GSTK in the AD3 and AD5 groups; and the mRNA levels of GSTO in the AD3, AD4 and AD5 groups were significantly upregulated (*p* < 0.05). The mRNA levels of GPx1 in the AD2 and AD5 groups were significantly higher than those in the AD4 group (*p* < 0.05). The expression levels of CAT, GPx4 and GSTT1 were independent of dietary treatment (*p* > 0.05).

### 3.5. Histological Structure in the Intestine

**Figure 7:**
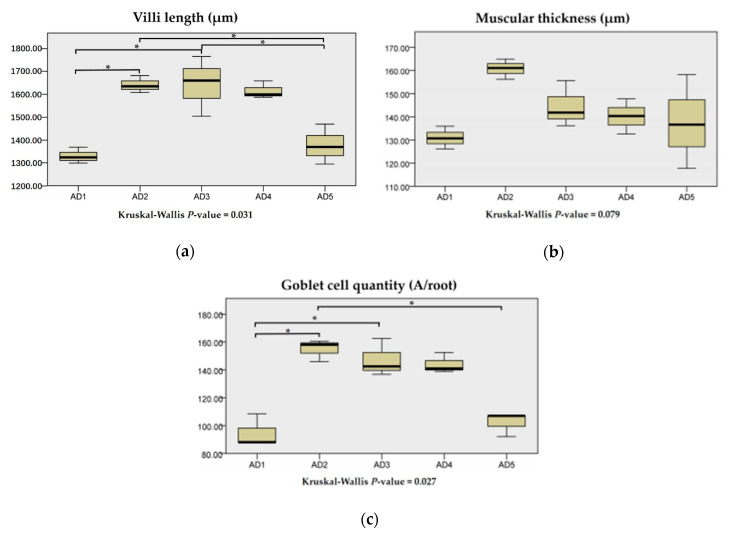
Effects of dietary andrographolide on intestinal morphology of rice field eel, *Monopterus albus*. (**a**), (**b**) and (**c**) are villi length, muscular thickness and goblet cell quantity, respectively. Note: * *p* < 0.05.

**Figure 8:**
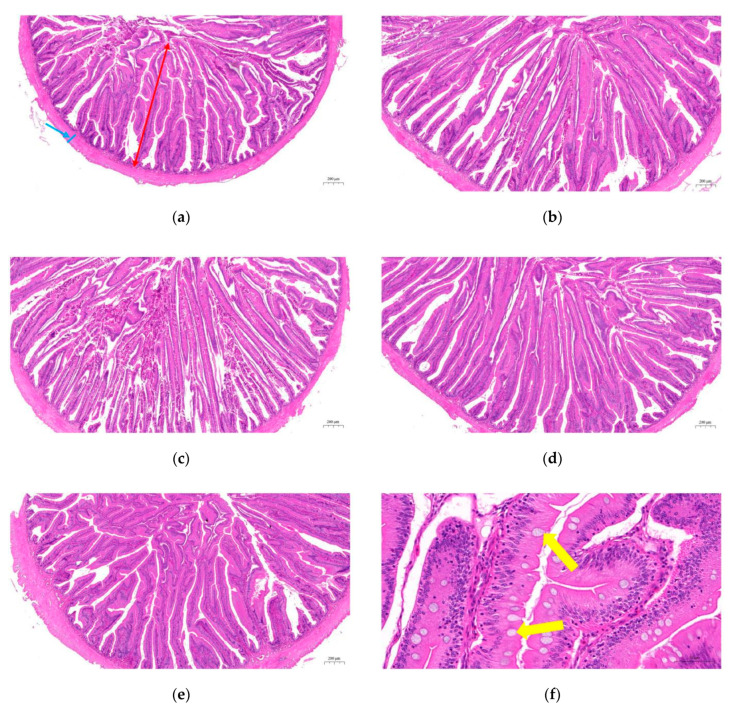
Effects of dietary andrographolide on intestinal histological structure of rice field eel (*Monopterus albus*). (**a**), (**b**), (**c**), (**d**) and (**e**) are AD1, AD2, AD3, AD4 and AD5, respectively (Magnification × 50). The magnification of (**f**) was 400. The red arrow indicates the villi length, the blue arrow indicates the muscular thickness and the yellow arrow indicates the goblet cell. The upper left is the scale bar.

As shown in Figure 7, villus lengths and goblet cell numbers in the AD2 and AD3 groups were significantly increased compared to the AD1 group (*p* < 0.05). Muscular thickness in the intestine showed no significant differences between the treatments, but the AD2 group was the highest (*p* > 0.05). In addition, the AD2, AD3 and AD4 groups showed a more orderly arrangement of intestinal villi (Figure 8).

### 3.6. Expressions of Physical Barrier-Related Genes in the Intestine

**Figure 9:**
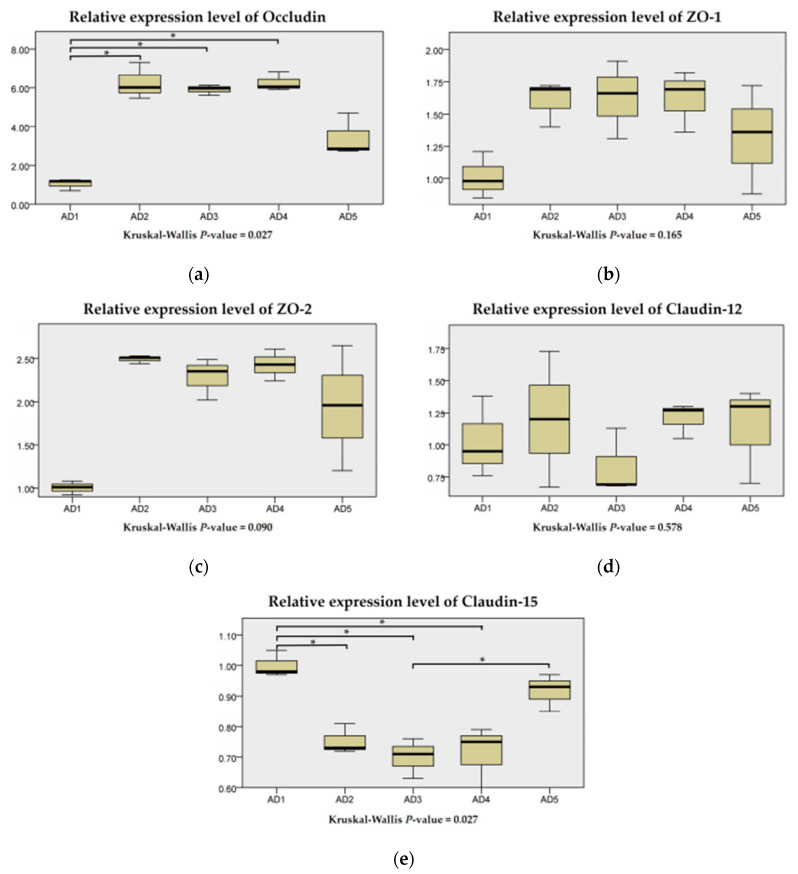
Relative expression levels of Occludin (**a**), ZO-1 (**b**), ZO-2 (**c**), Claudin-12 (**d**) and Claudin-15 (**e**) of rice field eel (*Monopterus albus*) fed diets containing graded levels of andrographolide. Note: * *p* < 0.05.

There were no significant differences in the mRNA levels of Claudin-12 among all treatment groups (*p* > 0.05; Figure 9). The mRNA levels of ZO-1 and ZO-2 were higher in the AD2, AD3 and AD4 groups than those in the AD1 group but not significant (*p* > 0.05). Compared to the AD1 group, the mRNA levels of Occludin in the AD2, AD3 and AD4 groups were significantly upregulated (*p* < 0.05), and the mRNA levels of Claudin-15 in the AD2, AD3 and AD4 groups were significantly downregulated (*p* < 0.05).

### 3.7. The mRNA Expression of Inflammation and the TLR Signaling Pathway in the Intestine

**Figure 10:**
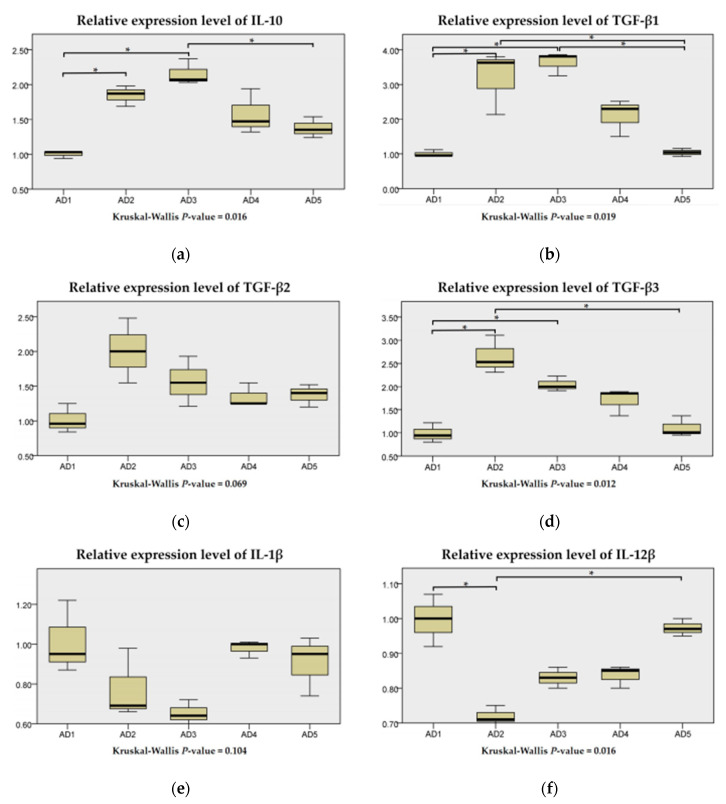
Relative expression levels of IL-10 (**a**), TGF-β1 (**b**), TGF-β2 (**c**), TGF-β3 (**d**), IL-1β (**e**) and IL-12β (**f**) of rice field eel (*Monopterus albus*) fed diets containing graded levels of andrographolide. Note: * *p* < 0.05.

As shown in Figure 10, the IL-10, TGF-β1 and TGF-β3 mRNA expression levels in the AD2 and AD3 groups were significantly upregulated compared to the AD1 group (*p* < 0.05), while the mRNA expression level of IL-12β in the AD2 group was significantly downregulated (*p* < 0.05). The TGF-β2 and IL-1β mRNA expression levels showed no significant differences between the treatments (*p* > 0.05). However, the TGF-β2 mRNA expression in the AD2 group was the highest, and the IL-1β mRNA expression level in the AD3 group was the lowest (*p* > 0.05).

**Figure 11:**
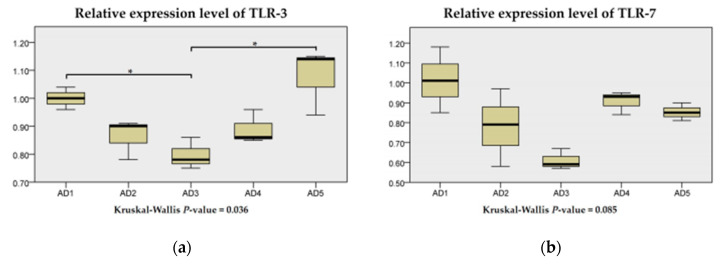

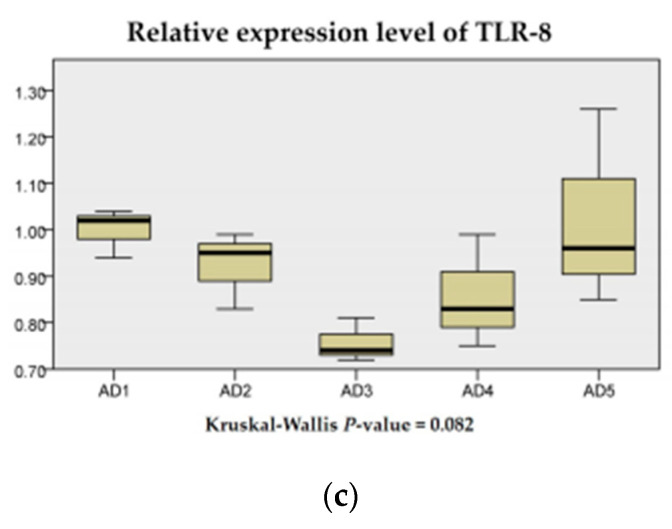
Relative expression levels of TLR-3 (**a**), TLR-7 (**b**) and TLR-8 (**c**) of rice field eel (*Monopterus albus*) fed diets containing graded levels of andrographolide. Note: * *p* < 0.05.

The TLR-3, TLR-7 and TLR-8 mRNA expression levels were the lowest in rice field eel fed the diet with 150 mg/kg andrographolide than those were fed other diets (Figure 11). The TLR-3 mRNA expression level in the AD3 group was significantly downregulated compared to the AD1 and AD5 groups (*p* < 0.05). With an increase in the andrographolide supplementation level, the TLR-3, TLR-7 and TLR-8 mRNA expression levels were first decreased and then increased.

### 3.8. Intestinal Microbiology Analysis

#### 3.8.1. Diversity Analysis

Compared to the AD1 group, the observed species, Chao1, ACE and PD whole tree indices were significantly decreased in the AD2, AD3 and AD5 groups (*p* < 0.05), and the Shannon index was significantly decreased in the AD2 and AD3 groups (*p* < 0.05; Table 3).

**Figure 12:**
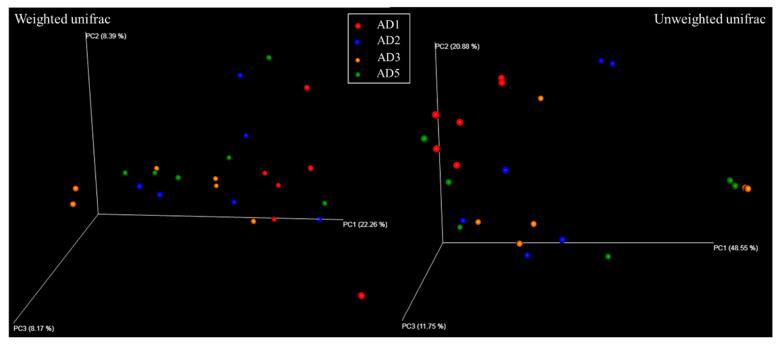
Principal coordinates analysis (PCoA) of the intestinal microbial community. Note: AD1 vs. AD2: R = 0.313, *p* = 0.009; AD1 vs. AD3: R = 0.313, *p* = 0.009; AD1 vs. AD5: R = 0.372, *p* = 0.005; AD2 vs. AD3: R = −0.042, *p* = 0.509; AD2 vs. AD5: R = 0.057, *p* = 0.293; AD3 vs. AD5: R = −0.115, *p* = 0.910.

PCoA based on the weighted and unweighted UniFrac metrics revealed a clear distinction in the microbiota, revealing considerable differences in the AD2, AD3 and AD5 groups compared to the AD1 group (Figure 12). ANOSIM analysis confirmed that the microbial community in the AD2, AD3 and AD5 groups differed significantly from that in the AD1 group (*p* < 0.05). Moreover, no significant difference was detected between the microbial communities in the AD2, AD3 and AD5 groups (*p* > 0.05).

#### 3.8.2. Microbial Composition

**Figure 13:**
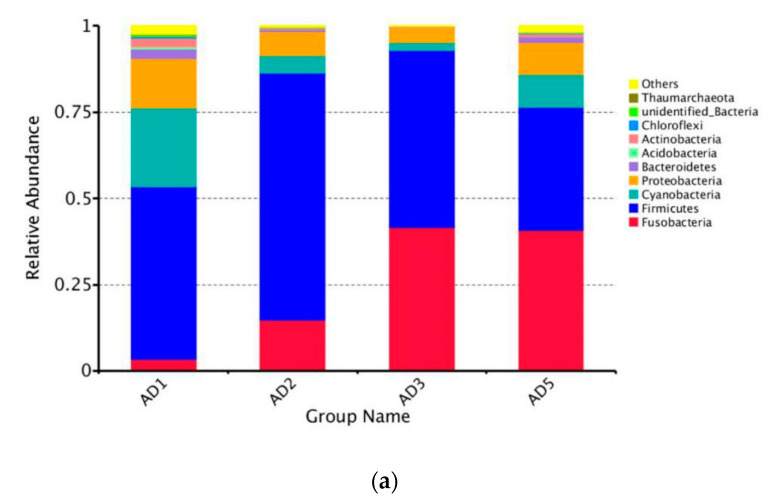

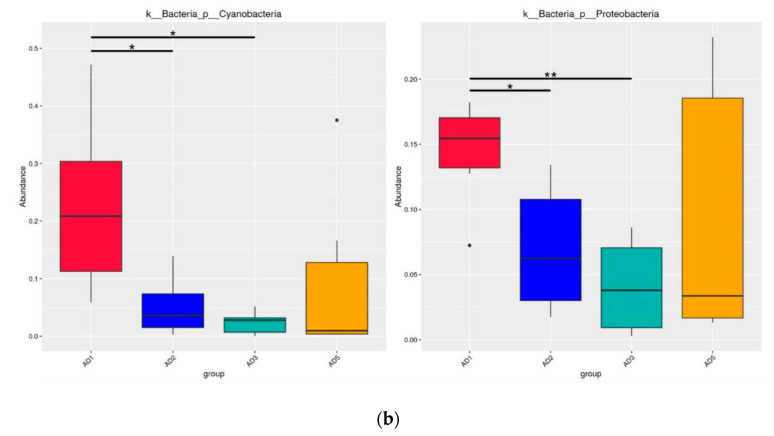
Relative abundance (**a**) of different bacterial phylum (Top 10) and box plots (**b**) showing significant variations of relative abundances of intestinal microbioma. Note: * *p* < 0.05, ** *p* < 0.01.

**Figure 14:**
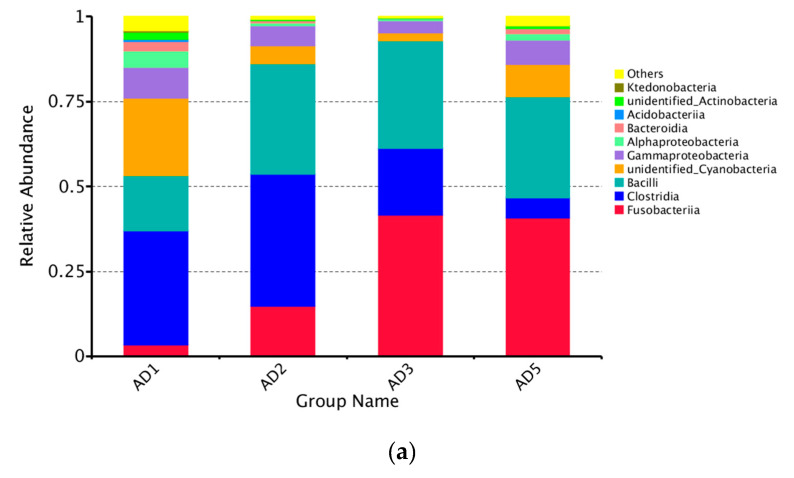

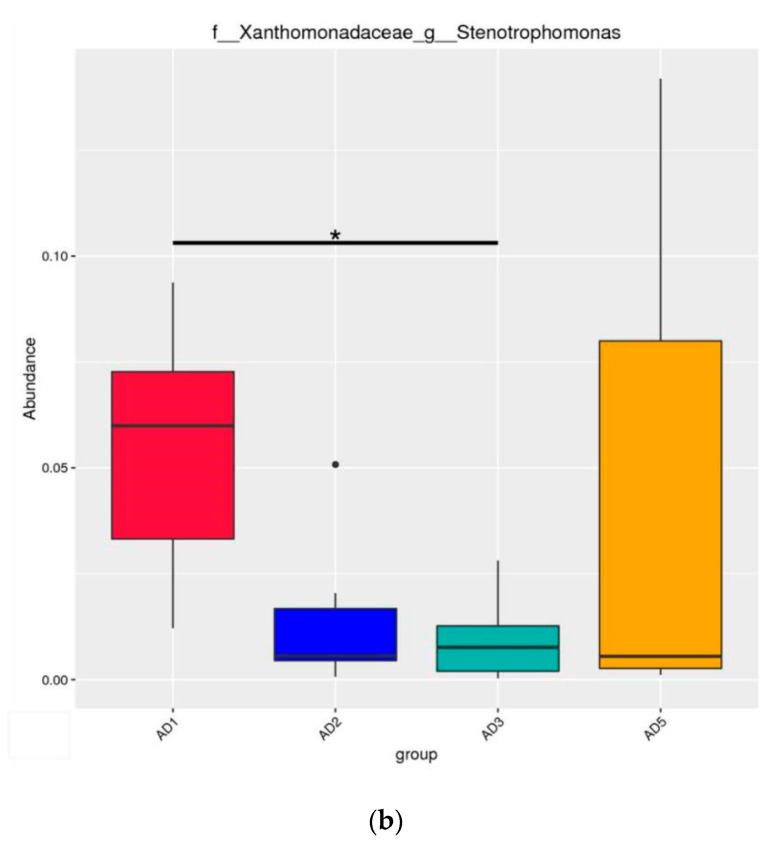
Relative abundance (**a**) of different bacterial classes (Top 10) and box plots (**b**) showing significant variations of relative abundances of intestinal microbioma. Note: * *p* < 0.05.

At the phylum level, the predominant members of the intestinal microbioma were Firmicutes, Fusobacteria, Cyanobacteria and Proteobacteria (Figure 13a). Among the experimental groups, the AD2 group exhibited the highest relative abundance of Firmicutes. The relative abundance of Cyanobacteria and Proteobacteria in the AD2 and AD3 groups significantly decreased compared to the AD1 group (*p* < 0.05) (Figure 13b). Specifically, Clostridia, Bacilli and unidentified Cyanobacteria predominated in the AD1 group, and Clostridia, Fusobacteriia and Bacilli predominated in the AD2, AD3 and AD5 groups (Figure 14a). Compared to the AD1 group, the AD2, AD3 and AD5 groups exhibited higher percentages of Fusobacteriia and Bacilli and lower percentages of unidentified Cyanobacteria, Gammaproteobacteria and Alphaproteobacteria. The relative abundance of Stenotrophomonas in the AD2 and AD3 groups was significantly lower than that in the AD1 group (*p* < 0.05; Figure 14b).

## 4. Discussion

A large number of documents have shown that herbs and natural plants improved growth performance, enhanced immunity and increased disease resistance in aquaculture [43,44,45]. The present study demonstrated that diets supplemented with 75–150 mg/kg andrographolide increased growth and decreased the feed conversion rate in *M. albus*. The fish growth is closely related to intestinal digestion and absorption capacity [46,47]. The intestinal morphological characteristics of fish include villus length, muscular thickness and goblet cell numbers. Villus length and muscular thickness are important indicators for measuring the efficiency of intestinal digestion and absorption [48]. In the present study, dietary andrographolide increased villus length, muscular thickness and the number of goblet cells. Another indicator that can reflect the ability to digest and absorb nutrients is intestinal digestive enzyme activities [49]. Our results showed that adding andrographolide to the feed enhanced the activity of digestive enzymes in the intestinal tract of *M. albus*. These data demonstrated that andrographolide improved the intestinal digestive and absorptive capacity, paralleled with the increased weight gain rate.

Unexpectedly, the weight gain rate of *M. albus* was lower than that of the control group when the content of andrographolide was over 150 mg/kg, although this difference was not significant. Consistent with the results of the present experiment, weight gain rate of Hamilton (*L*. *rohita*) [22] first increased and then decreased with increasing amounts of andrographolide. Because of the degeneration of the eel eye, the fish mainly depends on their sensitive sense of smell to find food [50]. Because andrographolide has a bitter odor [51], adding high concentrations of andrographolide to the diet will affect the feed intake of *M. albus*, leading to the decline in the growth performance.

Normally, ROS are produced during the metabolic progress [52]. When the ROS accumulate beyond the body scavenging ability, oxidative stress occurs [53]. To alleviate the oxidative stress of the body, fish have developed evolutionarily complete antioxidant enzyme systems (such as SOD, CAT, GPx, GR and GST), and antioxidants (such as GSH) to reduce the body’s redox [54,55]. Our results demonstrated that the addition of andrographolide enhanced the antioxidant ability, manifested by the decrease contents of ROS and MDA and the increased activities of SOD, CAT, GPx, GST, GSH, GPx and GR in the liver. A similar result was found that andrographolide can reduce the production of ROS in RAW264.7 cells [15]. In addition, andrographolide is used to counteract the damage to chondrocytes caused by H_2_O_2_. It has been found that andrographolide can reduce the oxidative stress injury of chondrocytes by increasing antioxidant enzyme activities (i.e., SOD and CAT) and reducing ROS in articular cartilage cells [56]. It is well known that the Nrf2/Keap1 signaling pathway regulates the mRNA expression levels of antioxidant enzymes genes [28,57]. In this study, it was found that the appropriate level of andrographolide significantly upregulated the mRNA levels of Nrf2 and its targeted genes SOD1, GSTK and GSTO in the liver. Keap1 inhibits nuclear translocation by binding to Nrf2, thereby inhibiting the antioxidant gene expression [58]. In this study, 150–225 mg/kg andrographolide significantly downregulated the mRNA levels of Keap1 in the liver, suggesting that the upregulated expressions of antioxidant genes of andrographolide may be related to the promotion of Nrf2 nuclear transposition through the reduction in Keap1 expression. However, how andrographolide affects the expression of these signaling molecules has not been reported, and further studies are needed.

Intestinal inflammation is a biological response, which is usually triggered by exogenous substances and products of tissue damage and characterized by the production of proinflammatory cytokines and the recruitment and activation of immune cells [59]. In turn, the produced cytokines play an important role in regulating intestinal inflammation in fish. The cytokines in aquatic animals mainly include proinflammatory cytokines (such as IL-1β and IL-12β, etc.) and anti-inflammatory cytokines (such as IL-10 and TGF-β, etc.) [60]. The upregulation of the proinflammatory factors (such as IL-1β), aggravated intestinal inflammation [61]. Studies have shown that andrographolide presents strong anti-inflammatory activity in mice [62]. In the present study, optimal andrographolide supplementation upregulated the mRNA expression levels of IL-10, TGF-β1, TGF-β2 and TGF-β3 and downregulated the mRNA expression levels of IL-1β and IL-12β in the intestine of *M. albus*, indicating the anti-inflammatory activity. Previous studies have demonstrated that TLRs convert the recognition of intestinal pathogen-associated molecules into signals involved in normal intestine maintaining inflammation under control [63]. TLRs are crucial pathogen recognition receptors in vertebrates. In this study, optimal andrographolide supplementation downregulated the mRNA expression levels of TLR-3, TLR-7 and TLR-8 in the intestine of *M. albus*. Previous studies have demonstrated that the activation of the TLR signaling pathway leads to increased proinflammatory cytokines (such as IL-1β) and decreased anti-inflammatory cytokines (such as IL-10) [64]. Therefore, this study indicated that the downregulation of proinflammatory cytokines by andrographolide might occur via suppression of the TLR pathway in the intestine.

The reduction in inflammatory response may be related to the improvement of the intestinal physical barrier and the prevention of pathogen invasion by andrographolide. The close junction of the fish intestinal tract is the first physical barrier to prevent pathogen invasion, which is very important to ensure the intestinal health of fish [65]. The tight junction proteins between fish cells are mainly divided into two major categories: cytoplasmic proteins (such as ZOs) and transmembrane proteins (such as Occludin and Claudins) [66]. Studies in fish have found that upregulation of occludin, ZO-1 and ZO-2 mRNA levels stabilize intercellular structural integrity, while upregulation of Claudin-12 and Claudin-15 mRNA levels disrupts the structural integrity of cells [67]. In this study, the addition of andrographolide significantly upregulated Occludin mRNA level and downregulated Claudin-15 mRNA level in the intestine of *M. albus*. Among other plant extracts, it has been found that the addition of sanguinarine upregulated Claudin, Occludin and ZO-1 mRNA levels in the intestine, and improved the barrier function of the tightly connected gut of *Ctenopharyngodon idellus* [38]. Therefore, these results indicated that andrographolide improved the intestine physical barrier by upregulating tight junction protein gene expression in *M. albus*.

The fish intestinal microbioma plays a crucial role in host health by stimulating the development of the immune system, enhancing in nutrient acquisition and competitively inhibiting opportunistic pathogens [68]; therefore, the microbioma is critical for intestinal health. Studies have shown that intestinal health is determined by complex microbial interactions and the interaction between the microbioma and the host intestinal system [69]. In addition, the establishment of a healthy intestinal microbioma is helpful for improving the production performance of aquatic animals. In this study, the control and experimental groups of *M. albus* shared the same dominant intestinal microbioma (including Firmicutes, Fusobacteria, Cyanobacteria and Proteobacteria). This result is similar to previous research in *M. Albus* [70]. Nevertheless, the addition of andrographolide was shown to distinctly increase the percentages of Fusobacteria and Firmicutes, concurrent with a significant decrease in those of Cyanobacteria and Proteobacteria. An increased proportion of Fusobacteria and Firmicutes is beneficial to intestinal health [71]. Proteobacteria are Gram-negative bacteria, and the outer membrane is mainly composed of lipopolysaccharides. Studies have shown that Proteobacteria represent a microbial signature of dysbiosis in the intestinal microbioma [72]. In addition, it was found at the genus level that the addition of andrographolide significantly inhibited the abundance of Stenotrophomonas. Stenotrophomonas are Gram-negative bacteria that are conditional pathogens [73]. These uncommon bacteria are highly antibiotic-resistant. The above results indicate that andrographolide has a certain improvement effect on the intestinal microbial composition of *M. albus*. In addition, studies have shown that high diversity and complex microbial communities contribute to the health of human and animal hosts [28,74]. However, compared with the AD1 group, the experimental groups (AD2, AD3 and AD5) showed significant decreases in the observed species Chao1, ACE and Shannon indices, which indicated that andrographolide can reduce the diversity of the intestinal microbioma. A possible reason for this finding is that andrographolide has a strong antibacterial effect that reduces the types of harmful bacteria in the intestinal tract of the fish and increases the richness of the dominant microbioma, thereby reducing species diversity [75]. Therefore, the addition of andrographolide to feed can increase the dominant microbioma and, thus, enhance the homeostasis of the intestinal microbioma.

## 5. Conclusions

Our results showed that the diets supplemented with andrographolide significantly improved growth performance, enhanced antioxidant capacity, regulated the intestinal physical barrier and microbioma of *M. albus*. Additionally, adding low-dose andrographolide (75 and 150 mg/kg) is more effective. In addition, dietary supplementation of andrographolide upregulated anti-inflammatory cytokines and downregulated proinflammatory cytokines in the intestine. The anti-inflammatory function of andrographolide may be related to the suppression of the TLR signaling pathway. These results can provide the valuable data for future rice field eel feeds.

## Figures and Tables

**Table 1:** Formulation and proximate composition of the experimental diets (dry weight).

Ingredients	g/kg	Proximate Composition	g/kg
Fish meal	360.0	Crude protein ^4^	431.6
Soybean meal	200.0	Crude lipid ^4^	51.6
Corn gluten meal	80.0	Ash ^4^	111.4
Soy protein concentrate	50.0		
Beer yeast	50.0		
Fish oil	20.0		
Choline chloride	5.0		
Premix ^1^	10.0		
Monocalcium phosphate	15.0		
α-starch	209.6		
Antioxidants ^2^	0.1		
Mold inhibitor ^3^	0.3		

^1^ Provided by Qingdao Master Biotechnology Co., Ltd. (Qingdao, Shandong, China). Vitamin and Mineral Premix composition (mg/kg diet): KCl 200 mg, KI (1%) 60 mg, CoCl_2_·6H_2_O (1%) 50 mg, CuSO_4_ 5H_2_O 30 mg, FeSO_4_·H_2_O 400 mg, ZnSO_4_·H_2_O 400 mg, MnSO_4_·H_2_O 150 mg, Na_2_SeO_3_·5H_2_O (1%) 65 mg, MgSO_4_·H_2_O 2000 mg, zeolite power 3645.85 mg, VB1 12 mg, riboflavin 12 mg, VB6 8 mg, VB12 0.05 mg, VK3 8 mg, inositol 100 mg, pantothenic acid 40 mg, niacin acid 50 mg, folic acid 5 mg, biotin 0.8 mg, VA 25 mg, VD 35 mg, VE 50 mg, VC 100 mg, ethoxyquin 150 mg and flour 2434.15 mg. ^2^ The antioxidant was butyl hydroxymethoxybenzene. ^3^ The mold inhibitor was sodium diacetate. ^4^ Crude protein, crude lipid and ash were measured values. Crude protein was determined using the Kjeldahl method and estimated by multiplying nitrogen by 6.25. Crude lipids were measured by ether extraction using the Soxhlet method. The crude ash assay was carried out by combustion in a muffle furnace at 550 °C for 16 h.

**Table 2:** Primers used for mRNA quantitative real-time PCR.

Gene	Forward Sequences (5′–3′)	Reverse Sequences (5′–3′)	Accession No. ^1^	PCR Efficiency (%)	Product Length
Occludin	TGTCGGGGAGTGGGTAAA	TCCAGGCAAATAAAGAGGCT	XM_020599328.1	97	130
ZO-1	GGCATCATCCCCAACAAA	GCGAAGACCACGGAACCT	XM_020621576.1	96	111
ZO-2	AGCCGAGGTCGCACTTTA	GCTTTGCTTCTGTGGTTGAT	XM_020615114.1	98	246
Claudin-12	TCACCTTCAATCGCAACG	ATGTCTGGCTCAGGCTTATCT	XM_020607277.1	99	250
Claudin-15	CTCGCTGCTTGCTTTGACT	TTGAAGGCGTACCAGGACA	XM_020611334.1	96	225
IL-10	TTTGCCTGCCAAGTTATGAG	CATTTGGTGACATCGCTCTT	XM_020593225.1	100	158
IL-1β	GAGATGTGGAGCCCAAACTT	CTGCCTCTGACCTTCTGGACTT	KM113037.1	97	127
IL-12β	CAAGTCAGTTGCCAAAATCC	CCAAGCAGCTCAGGGTCT	XM_020594580.1	99	103
TGF-β1	AACCCACTACCTCACTACCCG	GCCGAAGTTGGAAACCCT	XM_020605575.1	96	128
TGF-β2	ATTACGCCAAGGAGGTGC	GGGTTTTGAAGACGGAAGAT	XM_020622328.1	98	178
TGF-β3	AGTTTGTCGCTATCCACTTGC	GATGAGTTCCTTGGTGCTGTTA	XM_020590885.1	95	180
TLR-3	TATTTAGAGCCATACAGGG	CACAATCAAGAACGCACA	XM_020614353.1	100	244
TLR-7	ATCCTCACGACTTCCCTC	TTTCTTTCATCACCCACT	XM_020596482.1	97	205
TLR-8	AAGTGAAGCAGGATGAAG	AAGTCCCAGATTGAGTGA	XM_020596483.1	96	139
Nrf2	CTTCAGACAGCGGTGACAGG	GCCTCATTCAGTTGGTGCTT	XM_020596409.1	96	260
Keap1	AGCCTGGGTGCGATACGA	CAAGAAATGACTTTGGTGGG	XM_020597068.1	98	198
SOD1	AGCTGGCTAAGTTCTCATTCAC	GCAGTAACATTGCCCAAGTCT	XM_020598413.1	99	227
CAT	GTCCAAGTCTAAGGCATCTCC	CTCCTCTTCGTTCAGCACC	XM_020624985.1	96	106
GPx1	GTTCACCGCCAAACTCTT	TTCCCATTCACATCTACCTT	XM_020607739.1	98	303
GPx4	ATTTATGACTTCTCAGCGACAG	CCTTCAGCCACTTCCACA	XM_020612291.1	100	325
GSTK	TTGATGTTCCCCTGCGTTAT	CACCTGCTCTACCTGCTTGTC	XM_020610780.1	97	131
GSTO	GGGAGAAATAAAGGTGAGGATG	CAGATGAGTTGACAAGGCAGTT	XM_020600427.1	99	199
RPL-17	GTTGTAGCGACGGAAAGGGAC	GACTAAATCATGCAAGTCGAGGG	XM_020587712.1	98	160

^1^ The accession numbers come from National Center for Biotechnology Information (NCBI).

**Table 3:** Effects of dietary andrographolide on alpha-diversity in the intestinal microbial of rice field eel, *Monopterus albus*.

Index	AD1	AD2	AD3	AD5
Observed species	453.33 ± 58.21 ^b^	276.67 ± 35.79 ^a^	195.5 ± 44.83 ^a^	279.83 ± 59.10 ^a^
Shannon	4.45 ± 0.37 ^b^	3.02 ± 0.54 ^a^	2.83 ± 0.39 ^a^	3.46 ± 0.49 ^ab^
Chao1	478.56 ± 64.49 ^b^	308.9 ± 30.79 ^a^	218.57 ± 48.57 ^a^	296.75 ± 58.01 ^a^
ACE	478.4 ± 66.62 ^b^	318.77 ± 29.11 ^a^	228.69 ± 46.86 ^a^	302.55 ± 57.53 ^a^
PD whole tree	55.86 ± 6.65 ^b^	38.71 ± 4.34 ^a^	28.13 ± 4.74 ^a^	37.39 ± 6.08 ^a^

Values are means ± SE of six replicates. Mean values with a different superscript within a row for a parameter are significantly different, (*p* < 0.05).
